# On the Medicinal Use of Phosphorous and Its Compounds

**Published:** 1869-10

**Authors:** 


On the Medicinal Use of Phosphorus and its Compounds.—We take the following extracts from an article, with the above caption, by John C. Thorowgood, M. D., published in the *Practitioner* for July, 1869 :

Since the discovery and isolation of the element phosphorus by Brandt, of Hamburg, in 1669, it has become the practice with physicians in this and other countries occasionally to prescribe this substance as a remedy in cases where some special stimulant to the nervous centres has seemed to be required. Thus we find that phosphorus has been administered in cases attended with great prostration of the vital powers, as in the latter stages of typhus fever, also in such chronic diseases of the nervous system as epilepsy, paralysis, melancholia, amaurosis, &c., occurring in debilitated subjects ; and there is good evidence to show that in many of these nervous affections the effect of phosphorus, properly administered, has been decidedly beneficial. * *

The well known fact that in cases where an unusual degree of wear and tear of the nervous system is being sustained it is common to find an excess of phosphatic matter excreted in the urine, while the individual becomes increasingly weak, nervous, and irritable, appears to show that exhaustion of nervous force is in some way connected with a rapid oxidation and excretion of phosphorus from the system.

Considering these points, we can see that there is reason in seeking to administer phosphorus as an internal medicine where we have reason to suspect that the nutrition of nervous matter may be failing from a loss of its right proportion of this very essential ingredient.

We give phosphorus for its restorative action over weak nerves, just as we give iron to nourish and restore blood that is weak and poor from lack of this constituent. * * *

Solid phosphorus, given in as small a dose as 1½ grain, acts as a poison, death seeming to take place in a gradual and painless way, with perfect retention of consciousness. There may be some vomiting, and the substances ejected appear luminous in the dark, as also does the stomach itself after death, when cut open in a dark place; but it is rare to find any marked inflammation of this organ: in the case of a bird poisoned by eating several grains of phosphorus, I could find scarcely a trace of inflammation anywhere in the digestive tract. In a case recorded by Casper, where a dose of 3 grains of phosphorus proved fatal to a lady in twelve hours, the body after death presented the extraordinary phenomenon of luminous vapor issuing from each of its outlets.

Analysis of the various tissues of animals poisoned by phosphorus has demonstrated the presence of phosphoric acid in unusual amount: this arises from the oxidation of the phosphorus in the body. Phosphoric acid is also increased in the urine of those who have taken any preparation of phosphorus. The action of phosphorus as a poison appears not to be due to any direct action on the nervous system, but to its preventing the assimilation of oxygen by the constituents of the blood; by thus checking oxidation it may cause the fatty degeneration of the liver so often met with in those who have been poisoned by phosphorus, and which is doubtless connected with the symptoms of severe icterus often seen in the patients before death.

For medical use there are solutions of phosphorus in ether and also in almond oil. * * *

Another very useful preparation of phosphorus is a pill, made by melting finely-divided phosphorus with fat and then covering the pill with an impermeable coating.

Pills that I have seen and used, made by Messrs. Savory and Moore, contain 1-40th of a grain of phosphorus in each pill. Both Dr. Radcliffe and Dr. Althaus speak favorably of the good effect of phosphorus, given thus in very small doses, as a valuable tonic in many chronic nervous maladies. * * *

M. Tavignot, in France, has long been in the habit of using phosphorus in the form of a pill, containing 1-70th of a grain, as a remedy in nervous, chlorotic and strumous affections. In some neurotic and paralytic affections of the muscles of the eyeball, and of the lachrymal nerve, M. Tavignot has used liniments of phosphorated oil with advantage; and, dropped into the eye, this oil is asserted after some month's use to have a solvent action on cataract. * * *

As a gradual tonic and restorer of failing nerve force I prefer the hypophosphite of soda or of lime to the potash salt, and either of these salts appears to me to answer all the purposes of pure phosphorus as an internal remedy, while, at the same time, they are more manageable and agreeable medicines. In cases of nervous depression and torpor, with at times shooting neuralgic pains; or, in other cases, numbness and deadness of the limbs, as from feeble circulation, the hypophosphites prove useful, and the lime or soda salt can be given according to the way in which the stomach may seem to bear the one better than the other. When anæmia is present, the citrate of iron can be added to the hypophosphite of soda, or else the syrup of the hypophosphite of iron, or of iron with quinine, can be employed; and either of these syrups will prove an active tonic, removing neuralgic pains, chest oppression, and languor of circulation in a very evident way. * * *

